# Loss of the tumor suppressor BTG3 drives a pro-angiogenic tumor microenvironment through HIF-1 activation

**DOI:** 10.1038/s41419-020-03248-5

**Published:** 2020-12-11

**Authors:** Yu-Che Cheng, Hsin-Yi Chiang, Shang-Jung Cheng, Hung-Wei Chang, Yi-Ju Li, Sheau-Yann Shieh

**Affiliations:** Institute of Biomedical Sciences, Academia Sinica, 128 Sec. 2, Academia Road, Taipei, 115 Taiwan

**Keywords:** Tumour-suppressor proteins, Oncogenesis

## Abstract

*B-cell translocation gene 3* (*BTG3*) is a member of the antiproliferative BTG gene family and is a downstream target of p53. Here, we show that senescence triggered by BTG3 depletion was accompanied by a secretome enriched with cytokines, growth factors, and matrix-remodeling enzymes, which could promote angiogenesis and cell scattering in vitro. We present evidence that at least part of these activities can be explained by elevated HIF-1α activity. Mechanistically, the BTG3 C-terminal domain competes with the coactivator p300 for binding the HIF-1α transactivation domain. The angiogenic promoting effect of BTG3 knockdown was largely diminished upon co-depletion of HIF-1α, indicating that HIF-1α is a major downstream target of BTG3 in the control of angiogenesis. In vivo, ectopic expression of BTG3 suppresses angiogenesis in xenograft tumors; and syngenic tumor growth and metastasis were enhanced in *Btg3*-null mice. Moreover, analysis of clinical datasets revealed that a higher *BTG3*/*VEGFA* expression ratio correlates with improved patient survival in a number of cancer types. Taken together, our findings highlight the non-autonomous regulation of tumor microenvironment by BTG3 while suppressing tumor progression.

## Introduction

Proper control of cell proliferation is a prerequisite for the maintenance of genome integrity. Normal human fibroblasts undergo replicative senescence due to progressive shortening of chromosome telomeres^1,2^. Premature senescence can be induced upon oncogene activation^3,4^ or by the loss of tumor suppressor proteins^5–8^. Notably, oncogene-induced senescent cells were found to secrete a spectrum of factors, collectively known as the senescence-associated secretome (SAS), involved in cell growth, cell migration, inflammation, and angiogenesis, among others^9,10^. These factors are suggested to play roles in tumor suppression, tissue repair, organismal aging, and paradoxically, tumorigenesis through cell-autonomous and cell–non-autonomous pathways^11,12^.

*B-cell-translocation gene 3* (*BTG3*) is a member of the BTG gene family, members of which share a conserved N-terminal domain, but have divergent C-terminal domains^13^. The conserved N-terminal domain of this family has been shown to interact with CAF1, thereby modulating mRNA deadenylation or cell proliferation^14,15^. We have previously shown that BTG3 is transcriptionally activated by p53 upon genotoxic assault^16^. BTG3 binds E2F1 to control cell proliferation and the G2 checkpoint^16^. Loss of Btg3 in mice was shown to promote lung cancer^17^, and downregulation of BTG3 was found in human cancers, including prostate cancer^7,18–21^. The tumor suppressor activity of BTG3 could be attributed to interaction with and negative regulation of AKT, which is frequently deregulated in cancers. Binding of BTG3 blocks AKT membrane translocation, thus suppressing downstream signaling, such as β-catenin nuclear accumulation and activation^22^. It is therefore unexpected that rather than promoting cell proliferation, depletion of BTG3 in normal human fibroblasts induces acute senescence, apparently out of a fail-safe mechanism^7^. This is due at least in part to de-repression of the *INK-4a* locus and induction of expression of p16 through AP1-mediated upregulation of the histone demethylase JMJD3^7^. The mechanisms by which this senescence is bypassed in BTG3-downregulated cells and whether the senescence has any impact on the surrounding microenvironment and neoplastic transformation remain unclear.

The transcription factor HIF-1α is involved in the hypoxia response as well as other hypoxia-independent pathways, such as the immune response and metabolic shift pathways^23–25^. HIF-1α forms a heterodimer with HIF-1β and binds DNA through the N-terminal basic helix-loop-helix domain. Hypoxia induces HIF-1α stabilization via regulation of a central oxygen-dependent degradation domain (ODDD). Its C-terminal transactivation domain mediates the expression of a plethora of genes participating in glucose metabolism, immune response, angiogenesis, and tumor progression^26,27^. The activity of HIF-1α is also regulated by post-translational modification, especially acetylation and hydroxylation. Hydroxylation at asparagine residues within the C-terminal domain by FIH-1 prevents the interaction between HIF-1α and its coactivator p300^28^; moreover, deacetylation by SIRT1 at K674 disrupts the interaction between p300 and HIF-1α^29^.

Given the diverse roles of cellular senescence, we wondered if loss of BTG3 may act as a “double-edge sword”. That is, on the one hand, it stops cell proliferation while being capable of promoting neoplastic progression in the context of additional genetic aberration. Here we show that concomitant with permanent growth arrest, these cells produce a secretome similar to that of SAS. We present evidence that highlights the involvement of HIF-1α and the molecular basis of its regulation by BTG3.

## Results

### Analysis of the secretome associated with BTG3-depleted normal human fibroblasts

**Fig. 1:**
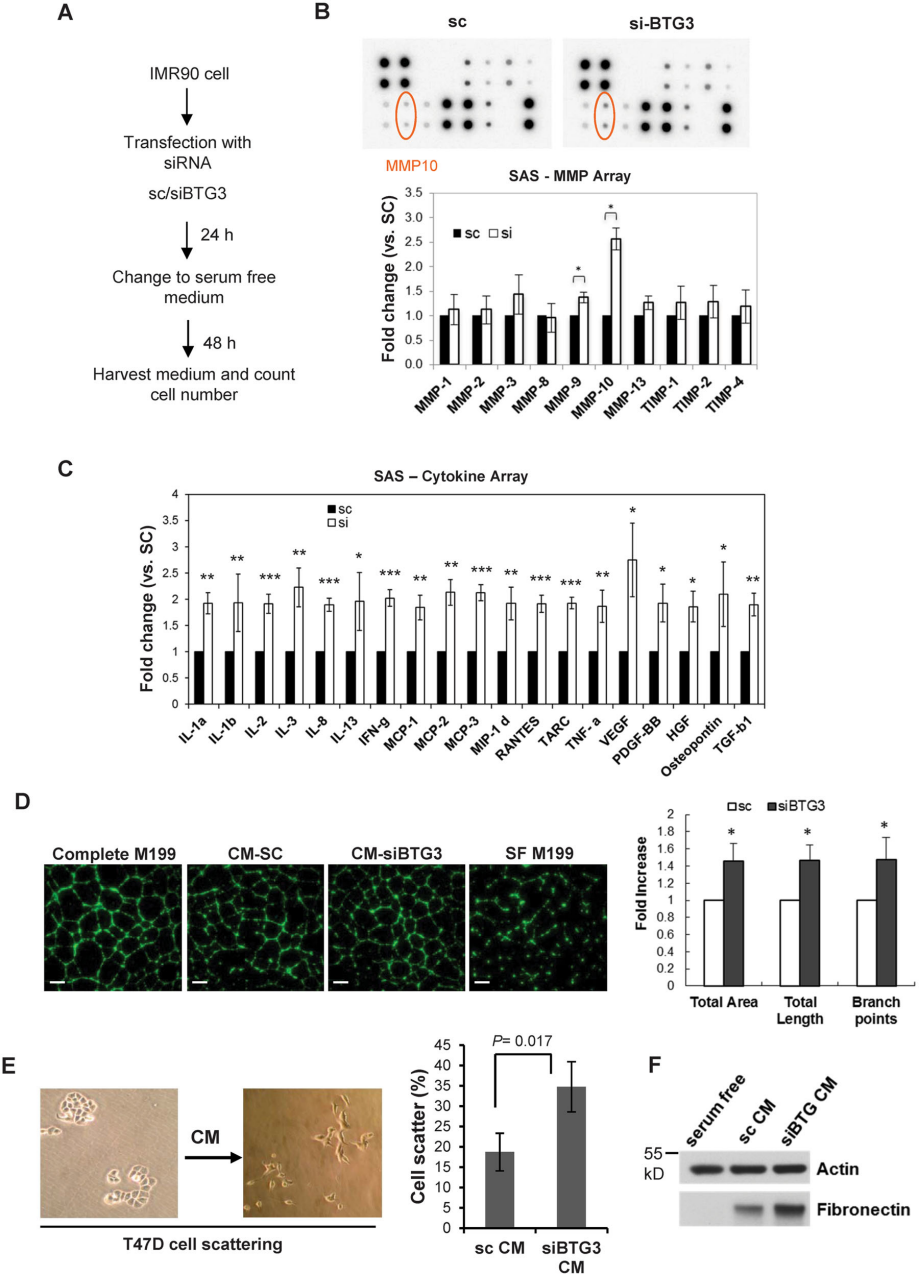
Characterization of the senescence-associated secretome (SAS) of BTG3-depleted IMR-90 cells. **a** Scheme for preparation of conditioned medium. **b** Analysis of MMP ELISA array with conditioned media from IMR90 cells transfected with control (sc) or BTG3 siRNA. Averages of two independent experiments are shown. **c** Analysis of the cytokine ELISA array with the same conditioned medium as used in (**b**). Mean ± SD from 3 independent experiments is shown. A complete set of data can be viewed in Fig. S1. **d** Conditioned medium (CM) from BTG3 knockdown IMR-90 promotes HUVEC tube formation. HUVEC cells were resuspended in CM and plated on Matrigel. After 16 h, cells were stained with Calcein AM, and the images were analyzed by MetaXpress High Content Image Acquisition & Analysis Software and the quantified results were shown on the left. Scale bar, 500 μm. **e**, **f** Medium conditioned by BTG3-depleted IMR-90 cells promotes scattering of T47D cells in vitro. T47D cells plated at low density were incubated with CM from control or BTG3 knockdown IMR90 cells for 48 h before scattering was measured (**e**). T47D cell lysates were also collected for western blot analysis using the antibodies indicated (**f**).

An increasing body of evidence points to the link between chronic inflammation and cancer progression. Pro-inflammatory cytokines, chemokines, and growth factors secreted by tumor-associated macrophages or cancer-associated fibroblasts have been shown to promote angiogenesis, cell migration, and invasion as well as to remodel the extracellular matrix to facilitate epithelial-to-mesenchymal transition (EMT)^10,30^. We have previously reported that RNAi knockdown of BTG3, a tumor suppressor^17,22^, induced acute senescence mediated by upregulation of the ERK-AP1-JMJD3-Ink4a signaling axis in the normal human fibroblast cell lines IMR-90 and WI-38^7^. To determine whether senescence induced by BTG3 depletion is associated with a secretome similar to the senescence-associated secretory phenotype (SASP)^9^ induced by oncogene activation, we analyzed the media conditioned by BTG3-knockdown cells using matrix metalloproteinase (MMP) and cytokine ELISA arrays. Cells were treated as indicated in Fig. 1a, and the results demonstrated those of the ten MMPs interrogated, only MMP-10 was significantly increased (Fig. 1b). Furthermore, various levels of increase were observed among the 80 cytokines/growth factors examined upon BTG3 depletion (Fig. S1), many of which are involved in chronic inflammation in vivo. Notably, several factors that were increased more than 2-fold, including SCF-1, VEGF, osteopontin, MCP-2/3, IL-3, IL-12, and IFN-γ (Fig. 1c), have all been implicated in various aspects of cancer development, and thus represent a unique signature of the secretome of BTG3-depleted normal human fibroblasts.

### Conditioned medium from BTG3-depleted fibroblasts promotes angiogenesis and cell scattering in vitro

Several factors shown to be elevated in our ELISA panels, including IL-6, IL-8, VEGF, and HGF, are known to promote angiogenesis and cell migration. We therefore performed an in vitro tube formation assay using human umbilical vascular endothelial cells (HUVECs) to determine whether the secretome of the BTG3-depleted cells would promote angiogenesis. Our results demonstrated that the conditioned medium (CM) from BTG3-depleted cells enhanced HUVEC tube formation by about 50% according to several parameters, including total tube length, area, and branch points (Fig. 1d). Furthermore, when incubated with the nonaggressive breast cancer cells T47D, the medium conditioned by BTG3-depleted cells promoted cell scattering, a hallmark of malignant progression (Fig. 1e). Concordantly, we also observed increased expression of fibronectin (Fig. 1f), a mesenchymal marker that has been linked to EMT. These results suggest that loss of BTG3 expression might shape a microenvironment that favors cancer development.

### Regulation of HIF-1 by BTG3

**Fig. 2:**
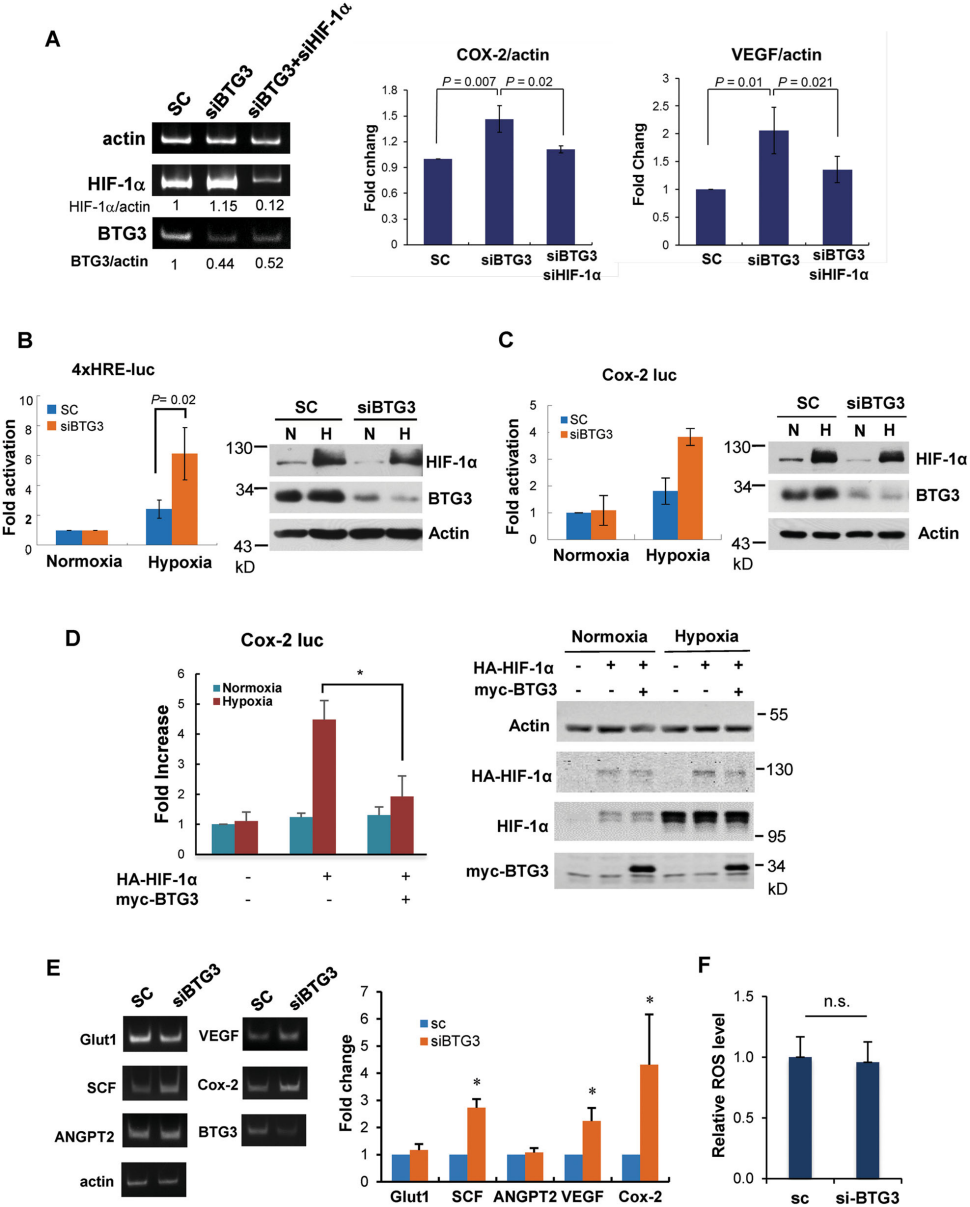
The activity of HIF-1 is increased by BTG3 depletion. **a** Expression of endogenous COX2 and VEGF was elevated upon BTG3 depletion in a HIF-1α-dependent manner. RNA was isolated from siRNA-transfected IMR90 cells and analyzed by RT-PCR. **b** Transcriptional activity of HIF-1α was enhanced in BTG3-downregulated cells. IMR-90 cells were first transfected with siRNA for 24 h then transfected with a luciferase reporter carrying four copies of the hypoxia response element (4×HRE). Cells were either left untreated or treated with hypoxia for 18 h prior to collection. All values were normalized to the cotransfected internal control. Western blots show the levels of BTG3 and HIF-1α under the treatment condition. **c** HRE in the COX-2 promoter was stimulated by BTG3 downregulation. Conditions were as in (**b**) but using the COX-2 reporter. **d** Overexpression of BTG3 suppressed the transcriptional activity of HIF-1α as assessed with the COX-2 reporter. **e** Selective induction of known HIF-1 targets in BTG3 knockdown IMR90 cells. SCF, but not Glu1 or ANGPT2, was induced by BTG3 downregulation, as assessed by RT-PCR. **f** BTG3 downregulation does not increase intracellular ROS.

While the BTG3 depletion-associated secretome may differ from the known SASP^11^ in its contents and levels, the possibility of NF-κB and HIF-1 activation was similarly implicated. Indeed, RNA analysis showed upregulation of several NF-κB and HIF-1 targets, such as *VEGFA, IL-8*, and *Cox2*, in BTG3-depleted IMR-90 cells, which was abolished upon either HIF-1α or RelA knockdown (Fig. 2a and Fig. S2A). These results suggest that BTG3 downregulation may activate the NF-κB and HIF-1 pathways either directly or indirectly.

To further assess the activity of NF-kB and HIF-1 in BTG3-depleted cells, we conducted reporter assays in IMR-90 cells. While the reporter driven by 4 copies of the HIF-1 response element (HRE) was upregulated in BTG3 knockdown cells (Fig. 2b), we were not able to detect any significant change in a reporter driven by the consensus NF-κB binding site (data not shown). BTG3 knockdown also showed no clear impact on p65 phosphorylation or IkB degradation (Fig. S2B). These results suggest that the effect on the NF-κB pathway may be indirect. Therefore, we directed our efforts in subsequent studies to focus on the HIF-1 pathway. In addition to the HRE reporter, the Cox2 reporter carrying the HIF-1 binding site in the *Cox2* promoter was also upregulated in BTG3 knockdown cells (Fig. 2c). Conversely, ectopic expression of BTG3 downregulated the Cox2 reporter (Fig. 2d), further confirming the involvement of BTG3 in the regulation of HIF-1. Among the endogenous HIF-1 targets examined by RT-PCR, we observed upregulation of SCF, VEGF, and Cox2 but not Glut1 or ANGPT2 (Fig. 2e), suggesting that they may be selective targets of BTG3.

To determine whether increased HIF-1 activity could be due to ROS elevation, which is often found in oncogene-induced or replicative senescent cells^31–34^, we measured and compared the ROS levels in control and BTG3 knockdown cells. The results indicated that BTG3 depletion did not alter cellular ROS levels (Fig. 2f), thus excluding its involvement in HIF-1 regulation in BTG3-depleted cells.

### Direct interaction between BTG3 and HIF-1α

**Fig. 3:**
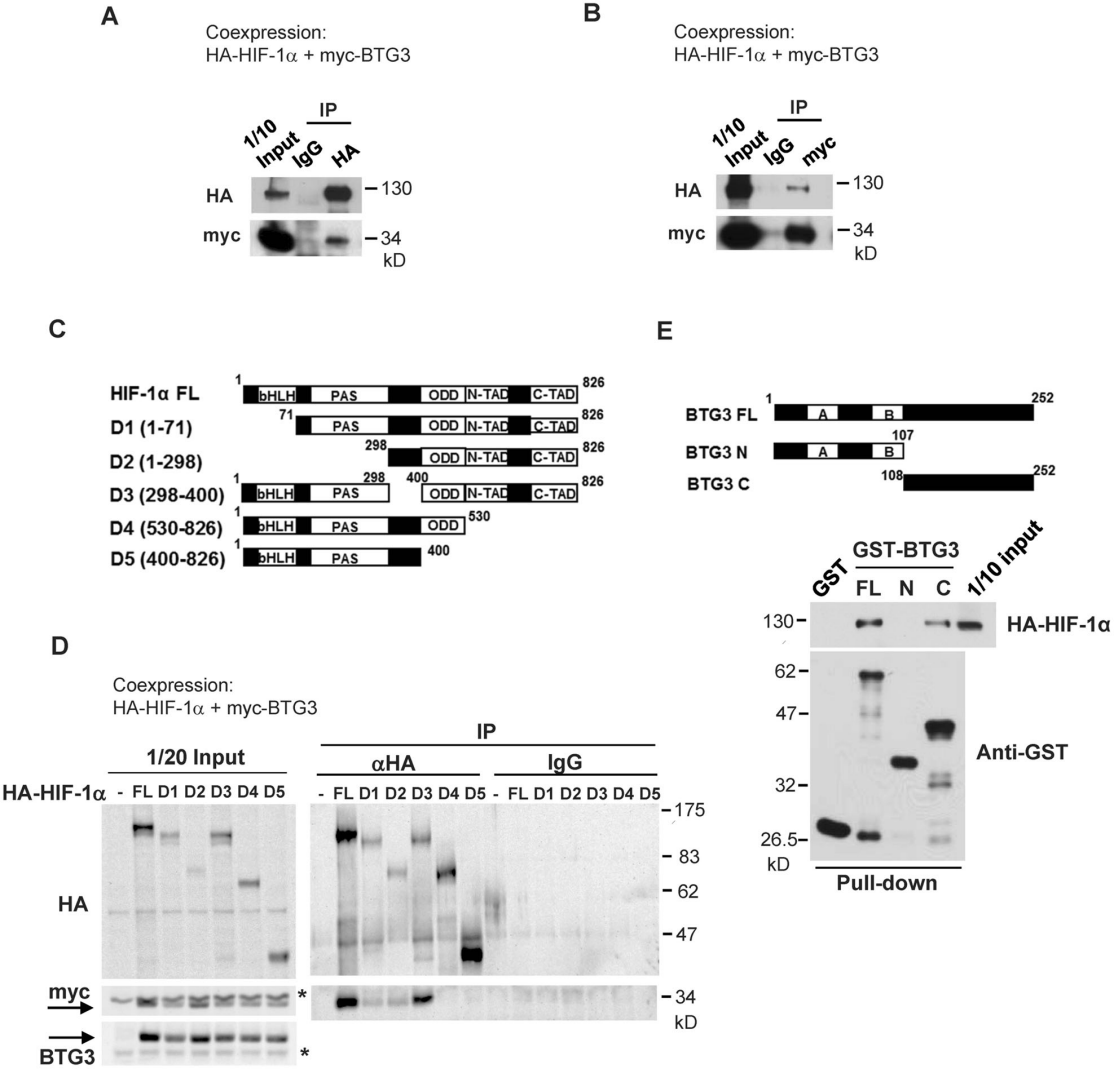
Interaction between BTG3 and HIF-1α. **a**, **b** Coexpression and coimmunoprecipitation of HA-HIF-1α and myc-BTG3. Lysates from transfected 293T cells were immunoprecipitated with anti-HA (**a**) or anti-myc (**b**). **c** Schematic diagrams of full-length (FL) HIF-1α and its various truncation or deletion mutants. **d** BTG3 binds the HIF-1α C-terminal transactivation domain. Co-immunoprecipitation was performed as in (**a**, **b**) with either FL HIF-1α or the indicated mutant. **e** Direct interaction of BTG3 with HIF-1α. GST pulldown assay was performed to show that recombinant BTG3 interacts with HIF-1α through the BTG3 C-terminal domain.

HIF-1 is composed of two subunits: the constitutively present HIF-1β and the labile, inducible HIF-1α. To understand how BTG3 affects the activity of HIF-1, the interaction between BTG3 and HIF-1α was explored. Co-expression and co-immunoprecipitation of HA-tagged HIF-1α and myc-tagged BTG3 in 293T cells indicated that they can interact in cells (Fig. 3a, b). The interaction domain in HIF1-α was further dissected with various truncation and deletion mutants (Fig. 3c, d). Results of co-immunoprecipitation indicated that the transactivation domain (amino acids 530–826) was responsible for interaction with BTG3, as evidenced by the loss of interaction in the D4 and D5 mutants (Fig. 3d).

Whether BTG3 and HIF-1α can interact directly was examined by in vitro GST pulldown assay. Our data showed that the full-length (FL) BTG3 and the BTG3 C-terminal domain, but not the N-terminal domain, could pull down HIF-1α overexpressed in lysates. This suggests that BTG3 can directly bind HIF-1α, which is most likely mediated through its C-terminal domain (Fig. 3e).

### Mechanisms underlying HIF-1α inhibition by BTG3

**Fig. 4:**
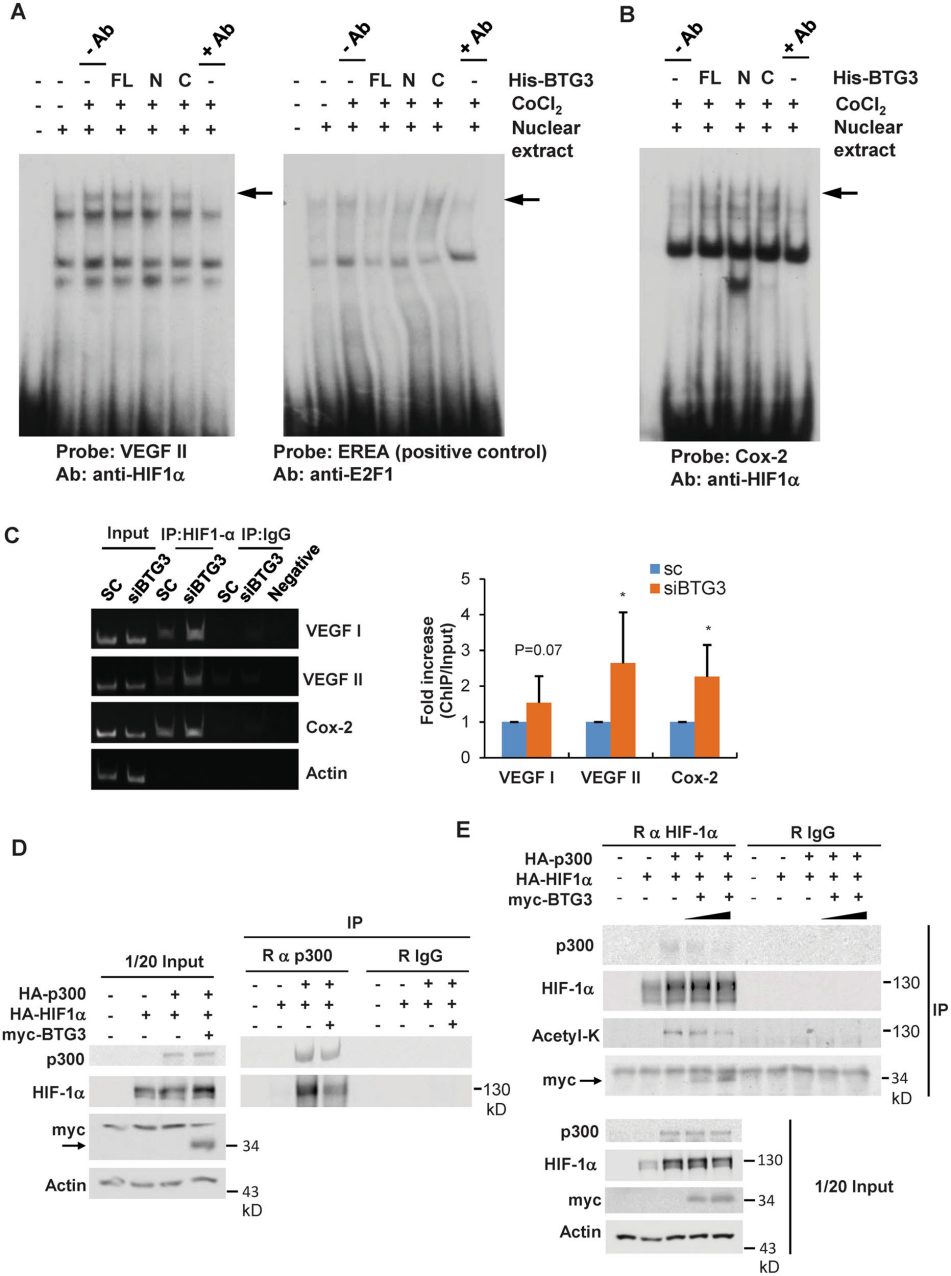
Mechanisms underlying HIF-1 inhibition by BTG3. **a** Binding of HIF-1α to HRE in vitro was not affected by BTG3. EMSA was conducted with nuclear extracts prepared from untreated cells or cells treated with CoCl_2_ and ^32^P-labeled oligonucleotide carrying HRE from the *VEGFA* promoter. Right panel, positive control showing that binding of E2F1 to the response element in the ARF promoter (EREA) is inhibited by recombinant full-length (FL) BTG3 and the N-terminal domain but not the C-terminal domain. Arrows indicate the specific protein–DNA complexes which could be competed away by specific antibodies. **b** Same as in (**a**) but with the HRE from the *COX2* promoter. **c** Binding of HIF-1α to the *VEGFA* and *COX2* promoters was enhanced in BTG3-downregulated cells. Chromatin immunoprecipitation (ChIP) was performed with control or BTG3 knockdown IMR-90 cells using anti-HIF-1α antibody. VEGFI and VEGFII represent two sequences in the *VEGFA* promoter that conform to HRE. Mean ± SD from 3 independent experiments is shown. *, *P* < 0.05. **d** Coexpressed BTG3 disrupted the interaction between HIF-1α and p300. 293T cells were transfected with HA-p300 and HA-HIF-1α with or without cotransfection with myc-BTG3. Co-immunoprecipitation was performed using anti-p300. **e** p300-dependent HIF-1α acetylation was reduced by co-expression of BTG3. Experiment was conducted as in (**d**) except that anti-HIF-1α antibody was used for immunoprecipitation.

To further dissect the molecular basis of HIF-1α inhibition by BTG3, we determined whether the DNA-binding activity of HIF-1 was inhibited by BTG3. IMR-90 cells were treated with CoCl_2_ to increase endogenous HIF-1 or left untreated, and nuclear extracts were prepared for in vitro electrophoresis mobility shift assay (EMSA). The results showed that while recombinant FL His-BTG3 disrupted binding of E2F1 to EREA^16^, it did not affect HIF-1 binding to the cognate site in the *VEGFA* promoter (Fig. 4a). HIF-1 binding to the consensus site in the *Cox2* promoter was also unaffected by recombinant BTG3 in EMSA (Fig. 4b). These results suggest that BTG3 does not directly impact the intrinsic DNA-binding activity of HIF-1.

Next, binding of HIF-1 to its target promoters in cells was investigated by chromatin immunoprecipitation assay (ChIP) using an anti-HIF-1α antibody. The results showed that, in contrast to what can be expected from data of in vitro binding, binding of HIF-1α to the *VEGFA* and *Cox2* promoters was increased in BTG3 knockdown IMR-90 cells (Fig. 4c). This result, which is consistent with the results of the elevated expression of these genes (Fig. 2e), could also suggest that factors involved in recruiting HIF-1 or stabilizing its promoter binding may be at play in cells.

CBP/p300 has been shown to be an important coactivator of HIF-1^35,36^, which also has a stabilizing effect on the cellular complex between HIF-1α and its DNA-binding partner HIF-1β^37^. As BTG3, like p300, binds to the transactivation domain of HIF-1α, the possibility that BTG3 may somehow block the interaction between p300 and HIF-1 was investigated by co-expression in 293T cells followed by co-immunoprecipitation. As shown in Fig. 4d, the interaction between HIF-1α and p300 was dampened upon co-expression of BTG3. This was also verified by reciprocal immunoprecipitation (Fig. 4e). Furthermore, consistent with the reduced interaction with p300, the acetylation of HIF-1α was also diminished with increasing amount of BTG3, while total protein levels were unaffected (Fig. 4e). No measurable interaction was detected between p300 and BTG3 (Fig. S3). Cumulatively, these data show that by interfering with the interaction between HIF-1α and p300, BTG3 exerts a negative regulatory impact on the induction of HIF-1 target genes.

### Enhanced HUVEC tube formation by BTG3 ablation is mediated through HIF-1α

**Fig. 5:**
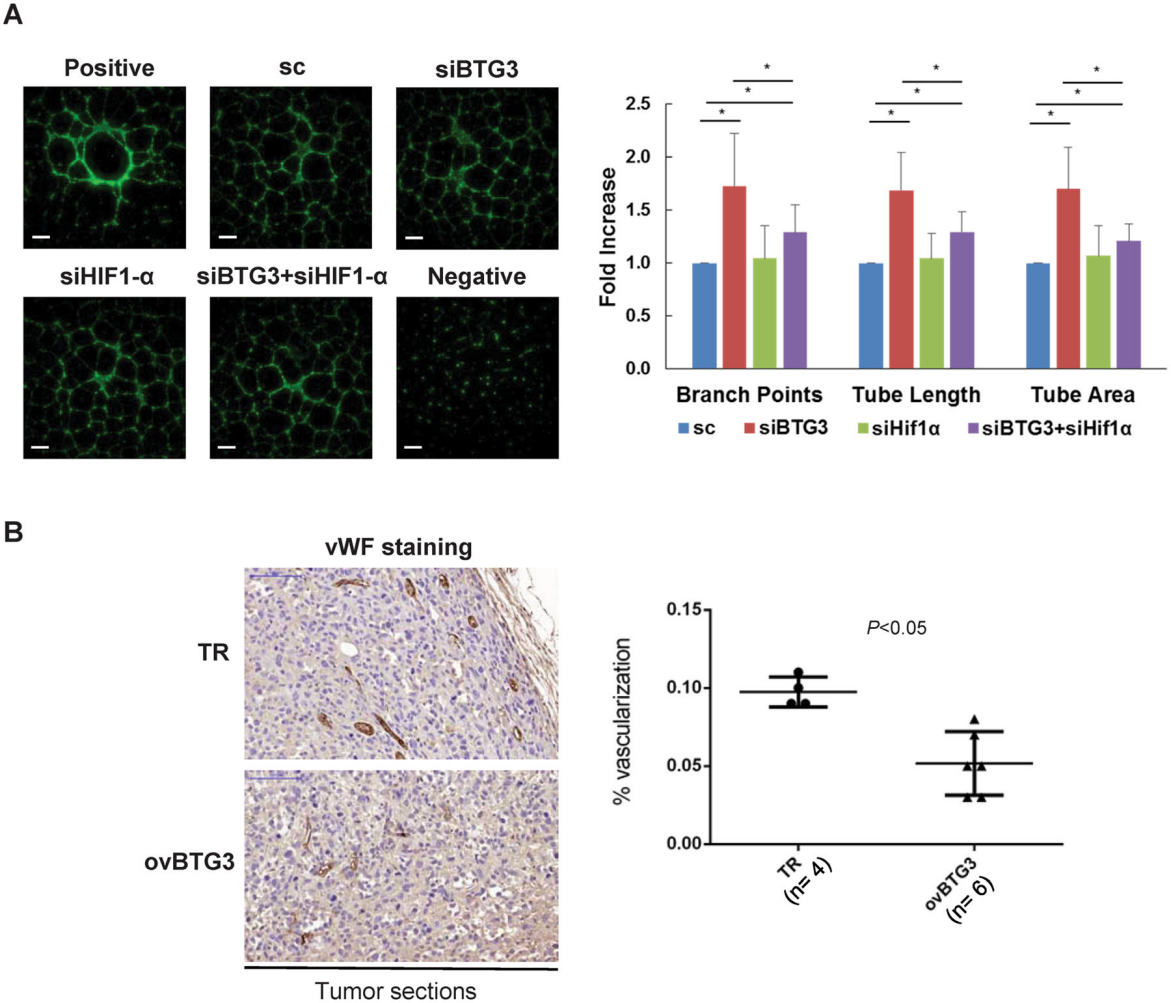
Regulation of blood vessel formation by BTG3 in vitro and in vivo. **a** HIF-1α is involved in the enhanced HUVEC tube formation conditioned by BTG3 knockdown. HUVEC tube formation assay was conducted with conditioned medium (CM) from IMR-90 cells transfected with control, BTG3 siRNA, HIF-1α siRNA, or both. Scale bar, 500 μm. Quantification was done as in (**d**). Mean ± SD from 3 independent experiments is shown. *, *P* < 0.05. **b** Overexpression of BTG3 inhibits angiogenesis in mouse xenograft tumor. Tumors were produced by injection of control (TR) or BTG3 overexpressing PC3 cells (ovBTG3)^22^. Angiogenesis was detected with immunohistochemical staining using the antibody recognizing the endothelial marker vWF. Scale bar, 100 μm.

As demonstrated above, BTG3 inhibits the activity of HIF-1 and CM from siBTG3 cells promoted HUVEC tube formation. We wondered whether the latter effect is mediated by elevated HIF-1 activity, which is known to promote angiogenesis along with other pathways^25^. To test this possibility, we simultaneously depleted BTG3 and HIF-1α in IMR-90 cells and collected CM to perform a HUVEC tube assay. The results showed that while siBTG3 CM promoted tube formation, co-depletion of HIF-1α (siBTG3 + siHIF-1α) largely abolished this effect (Fig. 5a), suggesting that the promoting effect of siBTG3 CM is attributable in most part to increased HIF-1 activity, likely through the expression of its downstream targets.

### BTG3 inhibits tumor angiogenesis in vivo

To determine if BTG3 could affect blood vessel formation in vivo, particularly during tumorigenesis, we compared the status of angiogenesis in mouse xenograft tumors with or without overexpression of BTG3^22^. Immunohistochemical staining for the endothelial marker von Willebrand factor (vWF) demonstrated that blood vessel formation was significantly reduced in BTG3-overexpressed tumors (Fig. 5b), thus supporting a negative regulatory role of BTG3 on tumor angiogenesis.

**Fig. 6:**
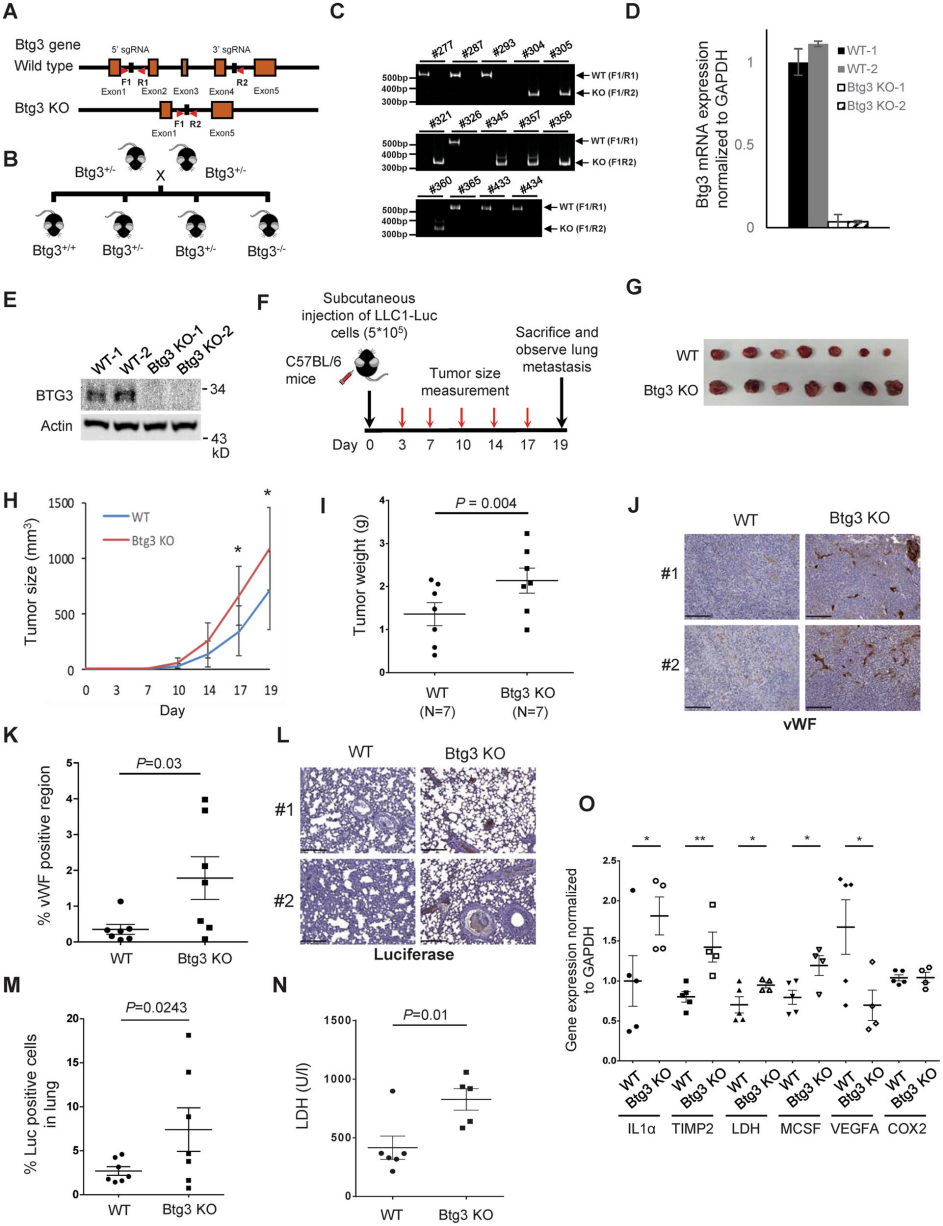
Genetic ablation of *Btg3* promotes syngenic tumor growth in mice. **a** Strategy of *Btg3* knockout in mice using CRISPR/Cas9. **b** *WT* and *Btg3*^*−*/*−*^ littermates produced by crosses between *Btg3*^+/*−*^ heterozygotes. **c** Genotyping by PCR of isolated tail DNA using either F1/R1 or F1/R2 primers. **d**, **e** Validation of Btg3 expression with qPCR (**d**) or western blot (**e**) using RNA or protein prepared from mouse liver. **f** Scheme for generation of syngenic tumor from LLC1-Luc cells. **g** LLC1-Luc tumors isolated from *WT* or *Btg3*^*−*/*−*^ mice at the experimental endpoint. **h** Growth curve of the LLC1-Luc tumors in *WT* and *Btg3*^*−*/*−*^ mice. Tumor size increased significantly faster in *Btg3*^*−*/*−*^ than in WT mice (*n* = 7). **i** Tumors harvested from *Btg3*^*−*/*−*^ mice weighed more than those from *WT* mice. **j**, **k** Increased staining of the endothelial marker vWF in tumors from *Btg3* KO. IHC was performed with paraffin-embedded tumor sections using anti-vWF antibody (**j**). Areas stained positive were quantified and percent positive region representative of each tumor is plotted in (**k**). Scale bar, 200 μm. **l**, **m** Increased lung metastasis in *Btg3*^*−*/*−*^ mice, as evidenced by IHC staining for luciferase (L) and its quantification (M). Scale bar, 200 μm. **n** Serum LDH levels in tumor-bearing *WT* and *Btg3*^*−*/*−*^ mice. **o** Expression of selected genes in LLC tumors as assessed by qPCR.

To further validate the role of BTG3 in regulating tumor angiogenesis, we sought to engineer a *Btg3*-deficient tumor microenvironment by genetic ablation of *Btg3* in mice (Fig. 6a–c). The *Btg3*^*−/−*^ mice appeared to develop normally and were born roughly according to the Mendelian ratio. RNA and protein analysis of isolated liver validated the absence of Btg3 expression in these mice (Fig. 6d, e). We then injected luciferase-expressing Lewis lung carcinoma cells (LLC1-Luc) subcutaneously into either *WT* or their *Btg3*^*−/−*^ littermates and compare tumor growth (Fig. 6f). Results showed that tumors implanted in the *Btg3*^*−/−*^ background grew much faster (Fig. 6g, h) and to a larger size (Fig. 6i) compared with those in *WT*. Tumor sections from the *Btg3*^*−/−*^ mice also stained more positively with vWF antibody (Fig. 6j, k). The possibility of lung metastasis was also investigated by IHC staining of lung sections for luciferase (Luc) expression. As a result, more Luc-positive cancer cells were detected in tumor-bearing *Btg3*^*−/−*^ mice than in *WT* (Fig. 6l, m). Furthermore, serum LDH, one of the HIF-1α downstream targets, was found to be elevated in *Btg3*^*−/−*^ mice (Fig. 6n). By qPCR, we also detected increased expression of several pro-inflammatory cytokines and pro-angiogenic factors such as IL-1α, TIMP2, LDH, and M-CSF in tumors harvested from *Btg3*^*−/−*^ mice (Fig. 6o). However, the expression of VEGFA and Cox2 was not increased (Fig. 6o), suggesting that these two factors may not be involved in angiogenesis in these tumors. Collectively, these data indicate that loss of *Btg3* drives a pro-angiogenic and pro-tumorigenic microenvironment which could also possibly promote tumor metastasis.

### The ratios of *BTG3*/*VEGFA* gene expression dictate patient survival in human cancers

**Fig. 7:**
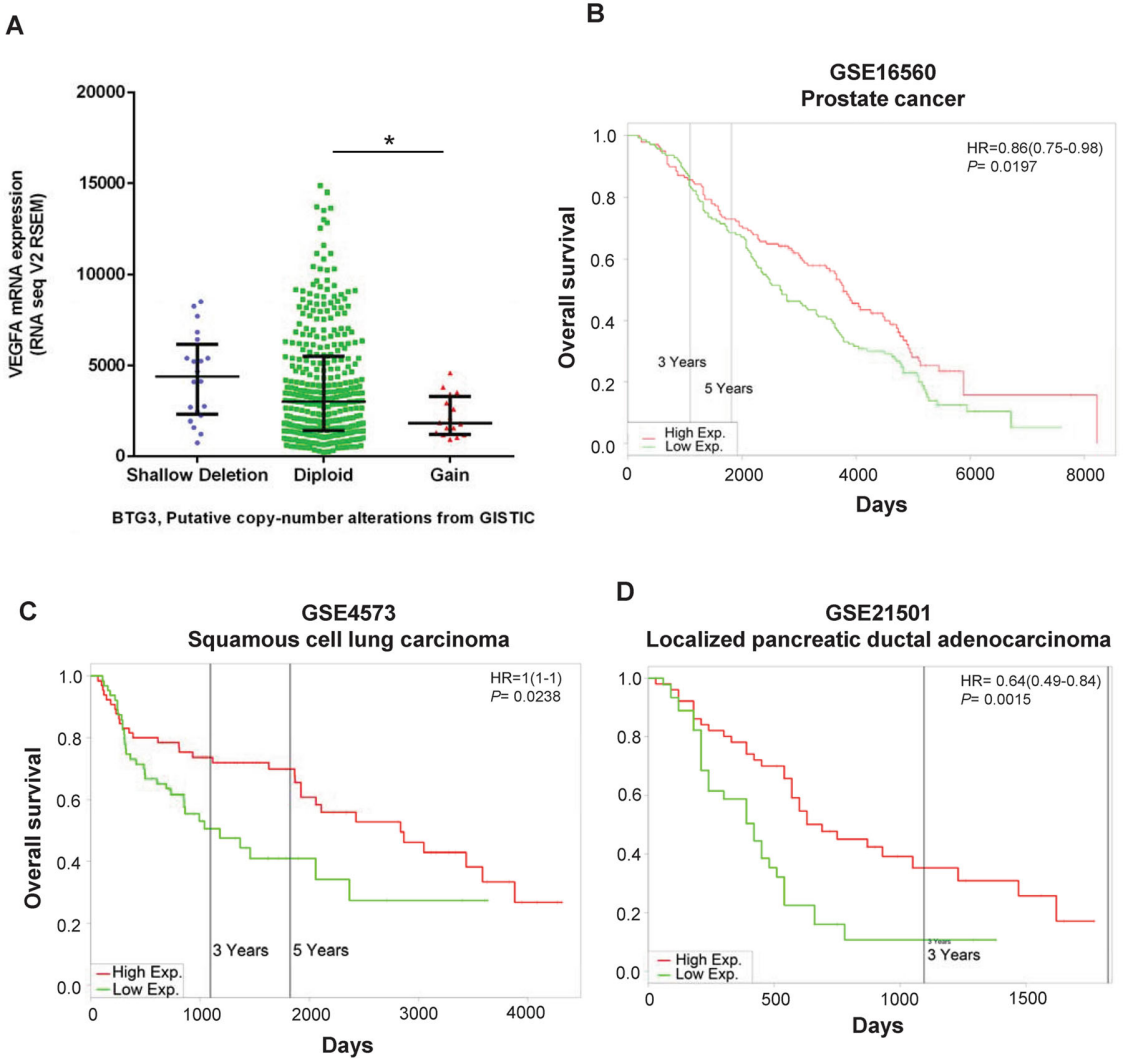
Ratios of *BTG3*/*VEGFA* expression are associated with patient survival in human cancers. **a** Copy number of the *BTG3* gene correlates inversely with levels of *VEGFA* expression in prostate cancer. The relationship between *BTG3* copy number and *VEGFA* mRNA expression was analyzed using cBioPortal for Cancer Genomics (http://www.cbioportal.org/) with data from TCGA for prostate cancer. Statistical analysis using one-way ANOVA shows marginal significance (*P* = 0.0703) among the three groups. The difference between diploid and copy number gain is significant when compared by unpaired *t* test (*P* = 0.035). **b**–**d** Low *BTG3*/*VEGFA* expression ratio predicts poor patient survival in prostate cancer (**b**), squamous cell lung carcinoma (**c**), and localized pancreatic ductal adenocarcinoma (**d**). GSE16560^39^, GSE4573^40^, and GSE21501^41^ were analyzed for their Kaplan–Meier overall survival in relation to the expression ratio of *BTG3*/*VEGFA*. The analysis was performed using the PROGgeneV2 platform (http://genomics.jefferson.edu/proggene).

To gain more insight into the relationship between BTG3 and tumor angiogenesis in human cancer, we analyzed available datasets in public domains using the PROGgeneV2 platform (http://genomics.jefferson.edu/proggene)^38^ for the association of patient survival and the relative expression of *BTG3* and *VEGFA*, a main downstream target of HIF-1 in angiogenesis. Interestingly, it was found that the expression of *VEGFA* is negatively correlated with the copy number of *BTG3* in prostate cancer (Fig. 7a). Patients with lower *BTG3*/*VEGFA* expression ratios had a poorer overall survival in prostate (Fig. 7b)^39^, lung (Fig. 7c)^40^, and pancreatic cancers (Fig. 7d)^41^. On the other hand, analysis of the same datasets did not support a relation of the *BTG3*/*HIF1A* ratios with patient survival in prostate and lung cancers, but showed a marginal effect in pancreatic cancer (Fig. S5), consistent with our result that BTG3 did not have an obvious impact on *HIF1A* RNA expression (Fig. 2a). Taken together, the results of these analyses further support a role of BTG3 in the regulation of tumor angiogenesis, whose loss may promote disease progression and dampen patient survival.

## Discussion

In this study, we identified HIF-1α as a target of regulation by BTG3 and elucidated the underlying mechanism and the implications on progression of human cancers. Importantly, we found that the effects could be mediated through non-autonomous paracrine of various pro-angiogenic and pro-inflammatory cytokines which together foster a pro-tumorigenic microenvironment; and this may partially explain the tumor suppressor role of BTG3. Thus, in addition to safeguarding genome stability through CHK1^42^ and keeping the AKT signaling at bay^22^, our findings indicate that BTG3 may also extend its grasp to control the cellular microenvironment through action on the transcription factor HIF-1α.

Still, there are a number of observations which highlight the complexity of cellular physiology. It is still unclear whether NF-kB is also directly regulated by BTG3 since many of the cytokines we observed are downstream targets shared by HIF-1 and NF-kB. Our protein analysis (Fig. S2B) and reporter assay (not shown) data would indicate that this is not likely. However, co-depletion of BTG3 and RelA had a dampening effect on the expression of its downstream targets (Fig. S2A), suggesting that NF-kB is somehow involved. One possible explanation is that cytokines such as IL-1 and TNF-α, upon induction by HIF-1, could activate NF-kB through an autocrine route, driving the expression of other canonical NF-kB targets. Evidence supporting this scenario includes the identification of an HIF-1 binding site in the *IL-1β* and *TNF-α* promoters^43–45^; and these cytokines are known to have an effect on NF-kB activation^46,47^. Although we have demonstrated direct regulation of HIF-1α by BTG3, this by no means excludes the involvement of other players.

One other issue concerns the seemingly discordant effect of BTG3 on the DNA-binding of HIF-1 in vitro and in cells. Although it is conceivable that binding of the activation domain would not affect the function of the DNA-binding domain, our in vivo binding data, assessed by chromatin immunoprecipitation, suggests otherwise. It seems that in the context of chromatin, the accessibility of the HIF-1 cognate site and the interaction with other factors could also have an impact. Studies by Ebert and Bunn^36^ pointed to the necessity of transcription factor binding sites adjacent to HRE and the requirement of p300 to interact with multiple transcription factors for high-affinity binding in vivo. In addition, interaction with p300 could also stabilize the complex between HIF-1α and its partner Arnt/HIF-1β, therefore increasing promoter binding in vivo^37^. Our data suggest the possibility that BTG3 competes with p300 to bind to the transactivation domain of HIF-1α, therefore reducing its promoter binding and transcriptional activity in cells. As cancer cells are frequently exposed to a hypoxic environment and are often under oxidative stress, it would be interesting to know how interaction between BTG3 and HIF-1α is regulated in normal, as opposed to cancer cells, under these conditions.

We have previously shown that normal human fibroblasts undergo acute senescence upon depletion of BTG3^7^. Surprisingly, *Btg3* knockout mice appeared normal, were not susceptible to tumor formation up to 2-years of age and are fertile, suggesting that loss of Btg3 alone in mice is not tumor-prone. Nevertheless, these mice upon old age did exhibit higher levels of selected cytokines and growth factors in serum reminiscent of the secretome of BTG3-depleted normal human fibroblasts (Fig. S4). And in line with the idea that chronic inflammation may promote tumor development^48^, here we showed that syngenic tumors progressed much faster and exhibited increased angiogenesis and metastasis in *Btg3*-null mice (Fig. 6g–m). Taken together, these data suggest that BTG3 can be a “landscaping” tumor suppressor, the loss of which may produce a microenvironment which favors tumor growth and spread. Whether these effects all resulted from HIF-1α deregulation or Btg3-deleted fibroblasts could be further debated. Although our in vitro and in vivo data support the idea that HIF-1α is a target of regulation, we cannot exclude the involvement of other direct or indirect regulation. Nevertheless, our study here has not only expanded the horizon controlled by the tumor suppressor BTG3 but also highlights the important role of a tumor suppressor in cell non-autonomous control of tumor progression.

## Materials and methods

### Cell lines

IMR90 normal human fibroblasts from ATCC were maintained in Eagle’s Minimum Essential Medium (Gibco, Life Technologies) supplemented with 10% fetal bovine serum (FBS; Invitrogen), 100 U/ml penicillin, 100 μg/ml streptomycin (Invitrogen), 0.1 mM nonessential amino acids, 2 mM l-glutamine, and 1 mM sodium pyruvate. HEK293T human embryonic kidney cells and mouse Lewis lung carcinoma cells (LLC1) were maintained in DMEM (HyClone) containing 10% FBS, 100 U/mL penicillin, and 100 μg/mL streptomycin. T47D breast cancer cells were grown in RPMI (Gibco, Life Technologies) with 10% FBS, 1 mM sodium pyruvate, and the same antibiotics as above.

LLC1-Luc cells were generated by transfection with pGL4.51 (Promega) followed by G418 (400 μg/ml; Gibco, 11811–031) selection. Drug-resistant cells were pooled and the expression of luciferase was verified by luciferase assay.

### RNA interference

Transfection of siRNA was conducted using Oligofectamine (Invitrogen). All siRNAs were synthesized by Sigma-Aldrich. The sequences targeted were: siBTG3, 5′-TTGAGAGGTTTGCTGAGAA-3′; siHIF-1α, 5′-GGGATTAACTCAGTTTGAA-3′; and siRelA, 5′-CCTTTCTCATCCCATCTTT-3′.

### MMP and cytokine array analysis

The cytokines and MMPs in conditioned media were analyzed using commercial antibody arrays (AAH-MMP-1 and AAH-CYT-5) according to the manufacturer’s instructions (RayBiotech, Inc). In brief, array membranes were incubated with the provided blocking buffer for 30 min, then 1 mL conditioned medium was added and incubated at 4 °C overnight. After thorough washing, membranes were incubated with biotin conjugated antibodies at 4 °C overnight, followed by incubation with HRP-conjugated streptavidin at RT for 2 h. Signals were detected by chemiluminescence. Intensity was normalized to internal positive controls for comparison.

For detection of cytokines in mouse serum, the Mouse Angiogensis Antibody Array C1 (AAM-ANG-1–4, RayBiotech) was used. One hundred μl of serum prepared from WT or Btg3-/- mice was incubated with the array according to the manufacturer’s instruction. Results were quantified using Metamorph.

### Tube formation assay

Human umbilical vascular endothelial cells (HUVEC) cells were obtained from Bioresource Collection and Research Center (BCRC) Taiwan and cultured in the manner suggested by ATCC. On the day of the experiment, cells were harvested and resuspended in serum-free, complete, or conditioned medium as a negative control, positive control, and experimental group, respectively. Twenty thousand cells were seeded in each well of a 96-well plate pre-coated with 50 μl Matrigel (BD Bioscience) in triplicate, and then incubated at 37 °C for 16 h. Cells were stained with Calcein AM (BD Bioscience) in PBS (4 μg/ml) at 37 °C for 30 min. Fluorescent images were captured by ImageXpress Micro Imaging XL System (Molecular Devices, USA), and analyzed by MetaXpress High Content Image Acquisition & Analysis Software version 5.0 (Molecular Devices, USA).

### Gene Expression

RNA was extracted from IMR-90 cells with TRIzol Reagent (Thermo Fisher Scientific, REF15596018), and reverse transcribed into cDNA using Oligo dT primer and SuperScript III reverse transcriptase (Invitrogen). The primers used for PCR are listed in Table S1. The PCR products were analyzed by gel electrophoresis and quantified using the Gene Tools Software (Syngene, USA).

For quantitative measurement of mouse gene expression, qPCR was performed using the primers listed in Table S2. RNA was extracted from tumors or mouse liver using TRIzol. Two μg purified RNA was reverse transcribed using SuperScript^®^ IV reverse transcriptase (Invitrogen, 18090010). The qPCR reactions were conducted in 50 μl in 96-well plate with the Power SYBR™ Green PCR Master Mix (Applied Biosystems, REF4367659). Reactions were carried out using the ABI 7500 system and relative expression values were calculated using ABI 7500 software. The housekeeping gene GAPDH was used to normalize expression.

### Constructs

HIF-1α cDNA was amplified from IMR90 by RT-PCR. The cDNA was subsequently cloned between the BamH I and Not I sites of the pXJN-HA or pXJN-myc vectors^16^ for mammalian expression. Various HIF-1α deletion constructs were generated by PCR and confirmed by sequencing.

### Luciferase reporter assays

IMR-90 cells were seeded and transfected the following day with indicative plasmids using Lipofectamine 2000 (Invitrogen). After incubation for 24 h, cells were treated with 50 μM CoCl_2_ for another 48 h. Luciferase assays were performed using the Promega Luciferase Assay System (E1501).

### ROS measurement

The generation of reactive oxygen species (ROS) was measured by flow cytometry using CM-H_2_DCFDA (C6827, Invitrogen) as a substrate. Briefly, cells were collected in full-serum medium, resuspended in PBS containing 5 μM CM-H_2_DCFDA and incubated for 30 min at 37 °C in the dark. Cells treated with H_2_O_2_ were used as a positive control. Samples were then analyzed using a FACSCalibur flow cytometer (Becton Dickinson).

### Co-immunoprecipitation

293T cells were plated on 35-mm dishes and transiently transfected by calcium phosphate precipitation. Cells were harvested 48 h after transfection in CSK buffer (10 mM PIPES pH 7.0, 100 mM NaCl, 3 mM MgCl_2_, 300 mM sucrose, 0.1% Triton-X-100) containing 10 mM NaF, 10 mM glycerophosphate, 1 mM sodium orthovanadate, 1 mM DTT, and protease inhibitors. After brief sonication, the cleared lysates were incubated with the antibody and protein G resins (Thermo Scientific), and rocked for 1.5 h at 4 °C. The beads were washed three times in the same buffer, boiled in the protein sample buffer, and analyzed by SDS-PAGE.

### Protein–protein interaction

GST pulldown assays were performed as previously described^22^ with recombinant GST-BTG3 proteins and 293T lysates expressing HA-HIF-1α.

### Electrophoretic mobility shift assay (EMSA)

The double-strand DNA probes were prepared by annealing the complementary oligonucleotides carrying the HIF-1 response element (HRE) in VEGF (II, 5′-CAGTGCATACGTGGGCTCCAACAGGTCCTC-3′) and COX-2 (5′- CAGTCTGTCCCGACGTGACTTCCTC-3′) promoters. The probes were labeled with [α-^32^P]dATP. Nuclear extracts were collected, incubated with recombinant His-BTG3 (full length, N-terminal, or C-terminal domain), and then with 30–50 ng salmon-sperm DNA as well as 2 μg BSA in DNA-binding buffer containing 20 mM Hepes (pH 7.9), 2 mM MgCl_2_, 0.1 mM EDTA, 10% glycerol, 2 mM spermidine, and 0.5 mM DTT for 30 min on ice. The labeled probe (2 × 10^4^ cpm) was then added to the reaction mixture and incubated for another 30 min on ice in a final volume of 20 μL. For the supershift assay, 200 ng of the rabbit-anti-HIF-1α antibody (100–134, Novus) was added. Protein–DNA complexes were separated on a 4% native polyacrylamide in 0.5x TBE at room temperature. The gels were dried before autoradiography.

### Chromatin Immunoprecipitation

The assay was performed as previously described^7^ using the anti-HIF1-α antibody (NB100-134, Novus Biologicals). For western blotting, the HIF-1α antibody used (610958) was sourced from BD Biosciences. The primers used for PCR are listed in Table S3. Amplification products were run on a gel and quantified using Gene Tools software (Syngene, USA).

### *Btg3* knockout mouse

Service provided by the Transgenic Mouse Models Core Facility, National Core Facility for Biopharmaceuticals Taiwan was employed. Briefly, *Btg3* knockout was generated in C57BL/6 mice by targeting sequences in intron 1 and intron 4 using CRISPR/Cas9. As a result, exons 2–4 were removed. Heterozygous ablated mice were crossed with *WT* mice for at least three generation before they were bred with each other to obtain *WT*, *Btg3*^*+/−*^, and *Btg3*^*−/−*^ littermates. Genotyping was completed by PCR analysis of tail DNA using the indicated primers: F1, 5′-CGGAGCTACGGTACCTGATT-3′; R1, 5′-CAGCGTTCCCTTTCAATTTG-3′; and R2, 5′-GTACTACTCTCTCTACTGCGA-3′.

### Tumor growth mouse model

All procedures were conducted according to the protocol approved by the Institutional Animal Care and Utilization Committee of Academia Sinica (AS IACUC, Protocol ID 11-12-271). For the tumor growth mouse model, 5 × 10^5^ LLC1-Luc cells were mixed with Matrigel (BD Bioscience) and were injected subcutaneously into the dorsal flank of 8-week old male *WT* or *Btg3* homozygous knockout C57BL/6 mice. Tumor size was measured regularly after tumor inoculation and was determined using the equation *V* = 0.5 × *a* × *b*^*2*^ (*a*, length; *b*, width in mm). When tumors reached approximately 1000 mm^3^ in size, mouse tumors and lungs were harvested after sacrifice. Tumor weight was measured at the same time.

### Immunohistochemistry

Tumors and lungs were removed from the mice, fixed in 4% paraformaldehyde overnight, and then switched to 50% ethanol before paraffin embedding. Antigen retrieval was performed with retrieval solution (Dako, S1700) at 95 °C for 20 min. Endogenous peroxidase activity was then blocked by incubation in 3% H_2_O_2_ (Sigma, 31642) at room temperature for 20 min. The sections were subsequently blocked in TBST (50 mM Tris pH 7.5, 150 mM NaCl, and 0.05% Tween20) containing 5% goat serum for 2 h at RT before incubation with anti-von Willebrand factor (VWF) antibody (Millipore, AB7356) or anti-luciferase antibody (Novus Biologicals, NB600-307) in 1% goat serum/TBST for 4 h at RT. After washing in TBST twice, samples were incubated with the Evision+ HRP-Polymer secondary antibody (K4002, Dako) for 1 h at RT. The staining was developed by the addition of diluted DAB substrate (K3467, Dako) and then counterstained in hematoxylin. Images were taken using a Zeiss Imager A1 microscope. For quantification, images were acquired and analyzed with Pannoramic 250 FLASH II software.

### Statistical analysis

Data from at least three experiments are expressed as mean ± SD. Unless specified otherwise, Student’s *t* test was used to analyze the data, and a difference was considered to be statistically significant if *P* < 0.05.

## Supplementary information

Supplemental Information
