# Supplementary material for: Loss of the tumor suppressor BTG3 drives a pro-angiogenic tumor microenvironment through HIF-1 activation
Source: Cell Death Dis. 2020 Dec 11;11(12):1046. doi: 10.1038/s41419-020-03248-5 (PMC7732837; doi:10.1038/s41419-020-03248-5)
Supplement: Supplementary file 1 — Supplemental Information [file 41419_2020_3248_MOESM1_ESM.pdf]

## Supporting Information

**Supplementary Table S1**

**Supplementary Table S2**

**Supplementary Table S3**

**Supplementary Figures and Figure Legends**

**Fig. S1** Related to Fig. 1

**Fig. S2** Related to Fig. 2

**Fig. S3** Related to Fig. 4

**Fig. S4** Related to Fig. 6

**Fig. S5** Related to Fig. 7

**Table S1.** Primers used in RT-PCR

| Genes | Primer Pairs (5′ to 3′) |
|---|---|
| **VEGF** | Forward: TGCAGATTATGCGGATCAAACC<br>Reverse: TGCATTCACATTTGTTGTGCTGTAG |
| **Cox-2** | Forward: TGCTGTGGAGCTGTATCCTG<br>Reverse: ACAGCCCTTCACGTTATTGC |
| **Glut1** | Forward: CTTCACTGTCGTGTCGCTGT<br>Reverse: TGAAGAGTTCAGCCACGATG |
| **ANGPT2** | Forward: TGCACAATGGTCTCACGTTCT<br>Reverse: GCAGCTCCCGTAAGTCAGAT |
| **SCF** | Forward: GTGCGTCAAAGAAAACTCAT<br>Reverse: TACTGCTACTGCTGTCATTC |
| **BTG3** | Forward: TCTTCCAATGTGGCACCCTT<br>Reverse: ATGTCACTGGAATTGGGCGA |
| **HIF-1a** | Forward: ACCACAGGACAGTACAGG<br>Reverse: TGGGGCATGGTAAAAGAAAG |
| **β-Actin** | Forward: CCAGAGCAAGAGAGGCATCC<br>Reverse: GTGGTGGTGAAGCTGTAGCC |

**Table S2. Primers used in qPCR (for mouse gene expression)**

| Genes | Primer Pairs (5′ to 3′) |
|---|---|
| **mTIMP2** | Forward: GCAGAAGGAGATGGCAAGA<br>Reverse: GATGCAGGCGAAGAACTTG |
| **mIL1a** | Forward: TCGTCAGGCAGAAGTTTGT<br>Reverse: CAACTCCTTCAGCAACACG |
| **mMCSF** | Forward: GGCAAGAGAAGTACCAGGGA<br>Reverse: ACTTGTAGAACAGGAGGCCC |
| **mLDH** | Forward: TTACACATCCTGGGCCAT<br>Reverse: CAGGAGTCAGTGTCACCT |
| **mVEGFA** | Forward: GGACAGACAGACAGACACC<br>Reverse: TCCTTCTCTTCCTCCCCTC |
| **mCOX2** | Forward: CCTGGTGAACTACGACTGC<br>Reverse: TTTAGTCGGCCTGGGATGG |
| **mBTG3** | Forward: ATGCGTTCATTGTTGCCAGC<br>Reverse: TTTCACGTCCACTTCCTTGC |
| **mGAPDH** | Forward: CAACTCCCACTCTTCCACCT<br>Reverse: CTTGCTCAGTGTCCTTGCTG |

**Table S3.** Primers used in chromatin immunoprecipitation assay

| Promoters | Primer Pairs (5′ to 3′) |
|---|---|
| **VEGF I** | Forward: TCTTTAGCCAGAGCCGGGGT<br>Reverse: GGACACACAGATCTGTTGGA |
| **VEGF II** | Forward: CTGGCCTCAGTTCCCTGGCA<br>Reverse: GTGGAGCTGAGAACGGGAAG |
| **Cox-2** | Forward: TGCTGCATATAGAGCAGATA<br>Reverse: TTCCTCTCCAGGAATCTGAG |
| **β-Actin** | Forward: CAACGCCAAAACTCTCCCTC<br>Reverse: TCGAGCCATAAAAGGCAACT |

**Supplementary figure legends**

**Figure S1.** Component characterization of medium conditioned by BTG3-depleted IMR-90 cells using cytokine ELISA arrays. Assays were performed as described in Fig. 1C with conditioned medium from control or BTG3-knockdown IMR-90 cells. Mean ± SD from 3 independent experiments is shown.

**Figure S2**. The effects of BTG3 depletion on gene expression is in part mediated through NFκB, possibly via an indirect route. (A) Co-depletion of RelA mitigated the induction of COX2 and IL-8 by BTG3 knockdown in IMR-90 cells. RNA was extracted at the time points indicated after siRNA transfection. RT-PCR was performed using the indicated primers. (B) NF-κB activation and IκB degradation was unaffected in BTG3 knockdown IMR-90 cells. Two days after siRNA transfection, cells were untreated or treated with TNFα (20 ng/ml) for the indicated time. Lysates were analyzed by immunoblotting using the indicated antibodies.

**Figure S3.** There is no apparent interaction between BTG3 and p300. Co-immunoprecipitation was performed using anti-myc antibody with lysates prepared from 293T cells transfected with myc-BTG3 and HA-p300.

**Figure S4.** Levels of selected cytokines are elevated in serum from *Btg3*$^{-/-}$ mice. Serum was prepared from aged *WT* or *Btg3*$^{-/-}$ mice (55 w), and angiogenesis-related cytokines and growth factors were detected using antibody arrays. The heatmap shows the results of quantification using Metamorph.

**Figure S5**. Relative expression of BTG3 and HIF1A in association with patient survival in human cancers. (A, B) The *BTG3*/*HIF1A* expression ratio is not associated

with patient overall survival in either prostate cancer (GSE16560) (A) or squamous cell lung carcinoma (GSE4573) (B). (C) High *BTG3*/*HIF1A* expression ratio is marginally associated with better patient survival in localized pancreatic ductal adenocarcinoma (GSE21501). DATA are analyzed using the ProgGeneV2 platform (http://genomics.jefferson.edu/proggene).

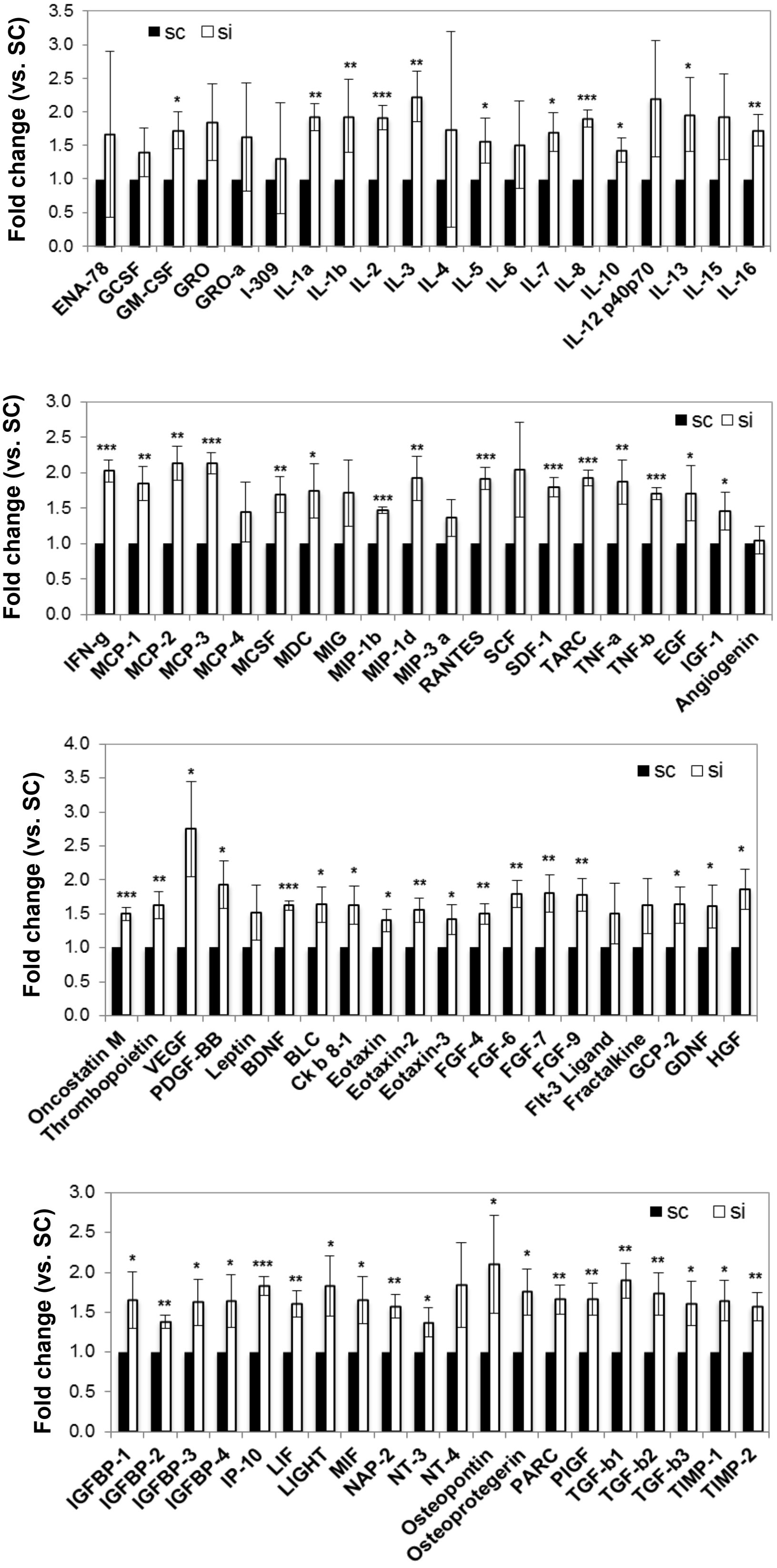
Fold change (vs. SC)
sc si
ENA-78 GCSF GM-CSF GRO GRO-a I-309 IL-1a IL-1b IL-2 IL-3 IL-4 IL-5 IL-6 IL-7 IL-8 IL-10 IL-12 p40p70 IL-13 IL-15 IL-16
IFN-g MCP-1 MCP-2 MCP-3 MCP-4 MCSF MDC MIG MIP-1b MIP-1d MIP-3 a RANTES SCF SDF-1 TARC TNF-a TNF-b EGF IGF-1 Angiogenin
Oncostatin M Thrombopoietin VEGF PDGF-BB Leptin BDNF BLC Ck b 8-1 Eotaxin Eotaxin-2 Eotaxin-3 FGF-4 FGF-6 FGF-7 FGF-9 Flt-3 Ligand Fractalkine GCP-2 GDNF HGF
IGFBP-1 IGFBP-2 IGFBP-3 IGFBP-4 IP-10 LIF LIGHT MIF NAP-2 NT-3 NT-4 Osteopontin Osteoprotegerin PARC PIGF TGF-b1 TGF-b2 TGF-b3 TIMP-1 TIMP-2

**Fig. S1**

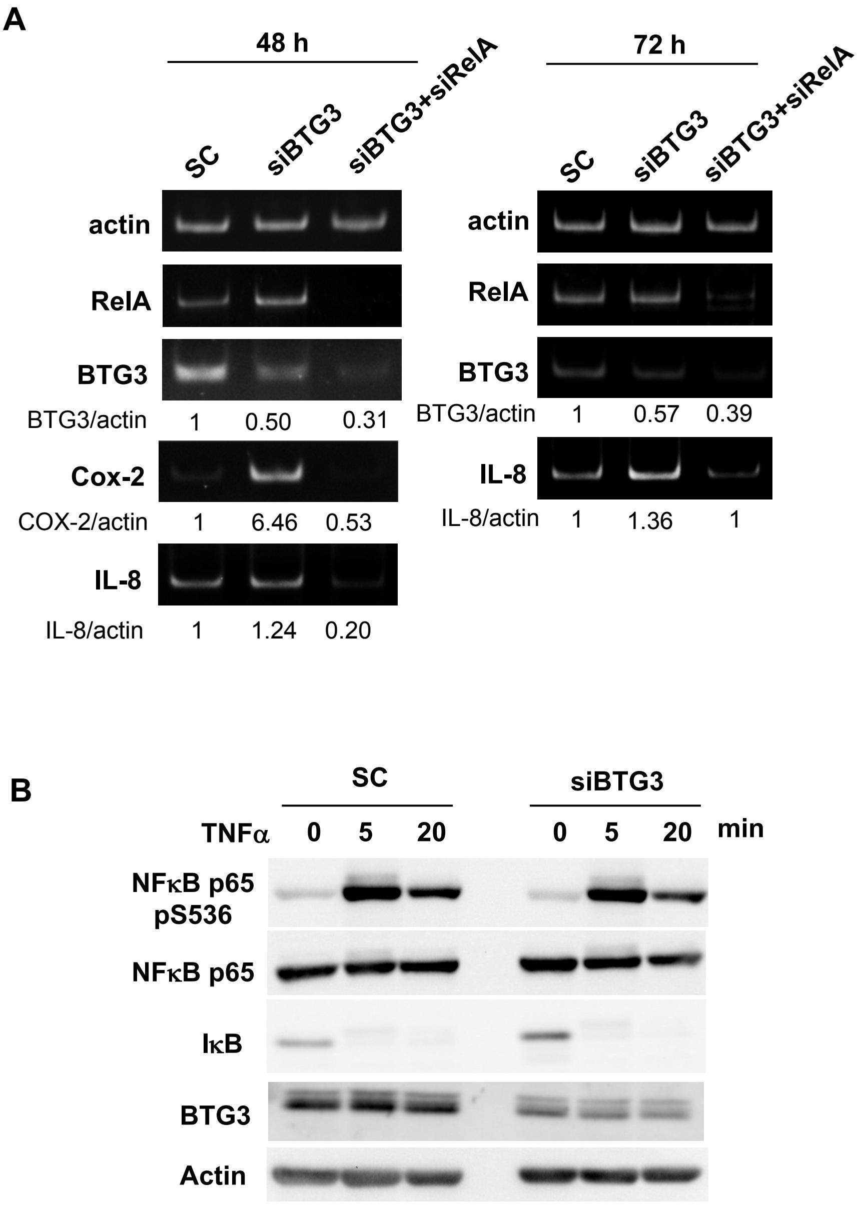
A
48 h
72 h
SC
siBTG3
siBTG3+siRelA
actin
RelA
BTG3
BTG3/actin 1 0.50 0.31
Cox-2
COX-2/actin 1 6.46 0.53
IL-8
IL-8/actin 1 1.24 0.20
BTG3/actin 1 0.57 0.39
IL-8/actin 1 1.36 1
B
SC
siBTG3
TNFα 0 5 20 0 5 20 min
NFκB p65 pS536
NFκB p65
IκB
BTG3
Actin



**Fig. S2**

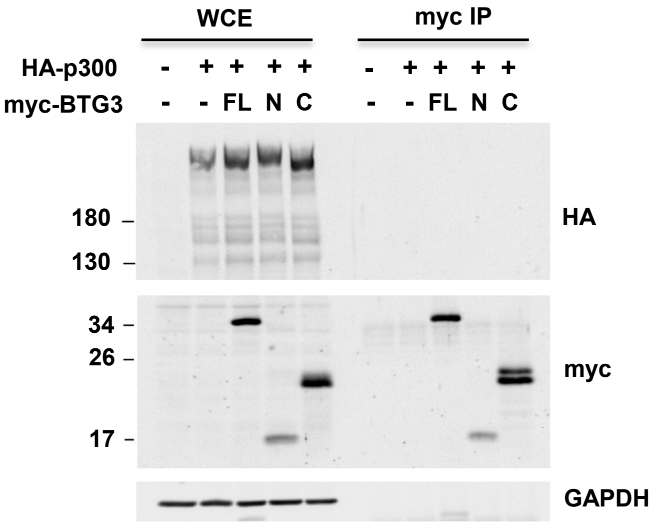


Fig. S3

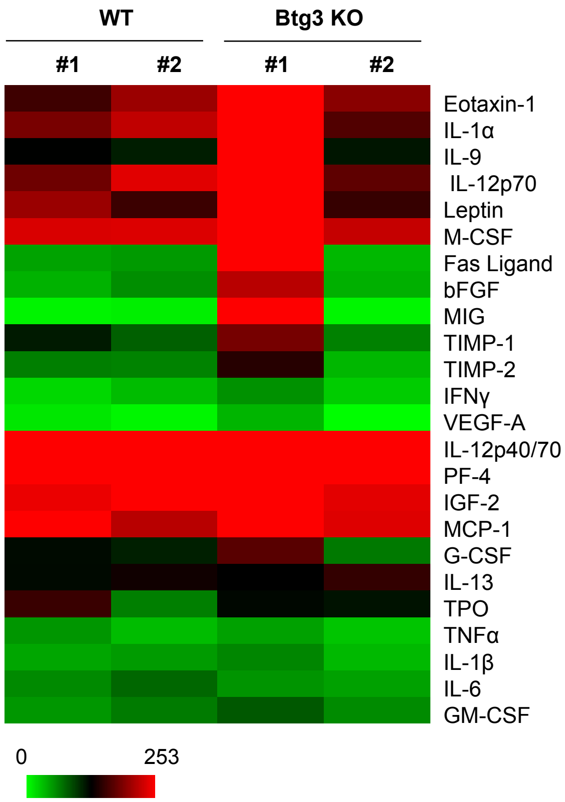
WT
Btg3 KO
#1
#2
#1
#2
Eotaxin-1
IL-1α
IL-9
IL-12p70
Leptin
M-CSF
Fas Ligand
bFGF
MIG
TIMP-1
TIMP-2
IFNγ
VEGF-A
IL-12p40/70
PF-4
IGF-2
MCP-1
G-CSF
IL-13
TPO
TNFα
IL-1β
IL-6
GM-CSF
0
253



**Fig. S4**

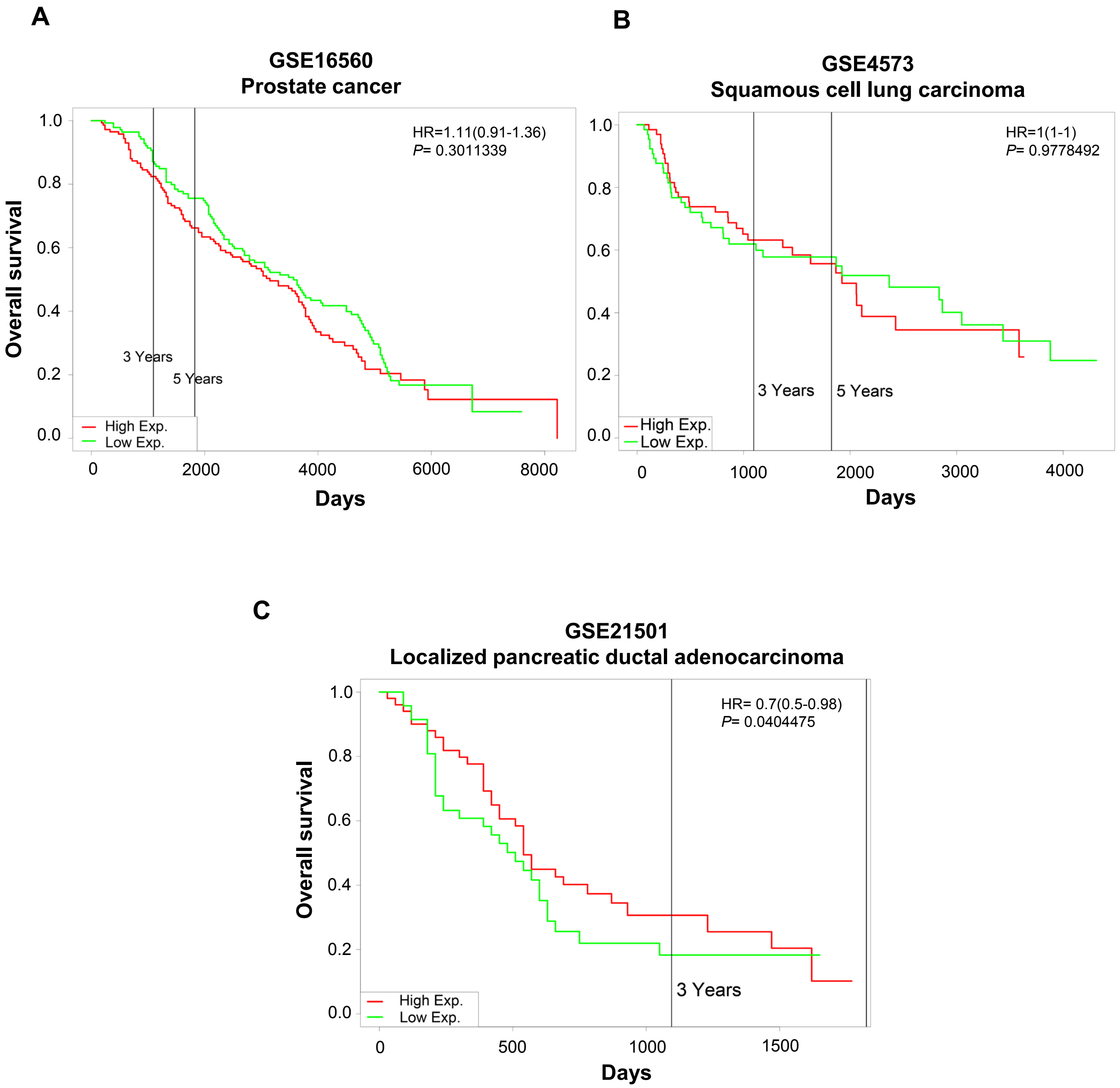

A
GSE16560
Prostate cancer
HR=1.11(0.91-1.36)
P= 0.3011339
3 Years
5 Years
High Exp.
Low Exp.
Overall survival
Days
B
GSE4573
Squamous cell lung carcinoma
HR=1(1-1)
P= 0.9778492
3 Years
5 Years
High Exp.
Low Exp.
Overall survival
Days
C
GSE21501
Localized pancreatic ductal adenocarcinoma
HR= 0.7(0.5-0.98)
P= 0.0404475
3 Years
High Exp.
Low Exp.
Overall survival
Days




**Fig. S5**
